# Perioperative Use of Intravenous Levodopa as an Anti‐Parkinsonian Drug: A Propensity Score Analysis

**DOI:** 10.1002/mdc3.13894

**Published:** 2023-10-13

**Authors:** Satoshi Kodama, Taisuke Jo, Hideo Yasunaga, Hiroyuki Ohbe, Nobuaki Michihata, Hiroki Matsui, Akira Okada, Yuichiro Shirota, Kiyohide Fushimi, Tatsushi Toda, Masashi Hamada

**Affiliations:** ^1^ Department of Neurology, Graduate School of Medicine The University of Tokyo Tokyo Japan; ^2^ Department of Health Services Research, Graduate School of Medicine The University of Tokyo Tokyo Japan; ^3^ Department of Respiratory Medicine, Graduate School of Medicine The University of Tokyo Tokyo Japan; ^4^ Department of Clinical Epidemiology and Health Economics, School of Public Health The University of Tokyo Tokyo Japan; ^5^ Department of Prevention of Diabetes and Lifestyle‐Related Diseases, Graduate School of Medicine The University of Tokyo Tokyo Japan; ^6^ Department of Clinical Laboratory Medicine, Graduate School of Medicine The University of Tokyo Tokyo Japan; ^7^ Department of Health Policy and Informatics Tokyo Medical and Dental University Graduate School of Medical and Dental Sciences Tokyo Japan

**Keywords:** Parkinson's disease, malignant neoplasm, overlap weighting

## Abstract

**Background:**

Perioperative discontinuation of oral anti‐parkinsonian medication can negatively impact the prognosis of abdominal surgery in patients with Parkinson's disease. Although intravenous levodopa may be an alternative, its efficacy has not yet been investigated.

**Objectives:**

To determine the efficacy of intravenous levodopa as an alternative to oral anti‐Parkinsonian drugs during gastric or colorectal cancer surgery.

**Methods:**

We identified patients with Parkinson's disease who underwent surgery for gastric or colorectal cancer between April 2010 and March 2020, using the Diagnosis Procedure Combination database, a nationwide inpatient database in Japan. Patients were divided into two groups: those who received intravenous levodopa during the perioperative period and those who did not. We compared in‐hospital mortalities, major complications, and postoperative length of stay between the groups after adjusting for background characteristics with overlap weights based on propensity scores.

**Results:**

We identified 648 patients who received intravenous levodopa and 1207 who did not receive levodopa during the perioperative period. In the adjusted cohort, the mean postoperative length of stay was 24.7 and 29.0 days (percent difference, −7.7%; 95% confidence interval, −13.1 to −1.5); in‐hospital death was 3.2% and 3.3% (adjusted odds ratio, 0.95; 95% CI: 0.54–1.67); and incidence of major complications were 21.4% and 19.3% (adjusted odds ratio, 0.89; 95% confidence interval, 0.70–1.13) in those with and without intravenous levodopa, respectively.

**Conclusions:**

Intravenous levodopa was associated with a shorter postoperative length of stay, but not with mortality or morbidity. Intravenous levodopa may improve perioperative care in patients with Parkinson's disease.

Parkinson's disease (PD) is the second most prevalent neurodegenerative disease affecting older adults.^1^ The mainstay of PD treatment is anti‐Parkinsonian drugs, which are usually administered orally at specific times of the day or at fixed intervals daily to keep the symptoms under control. Changes in the scheduling of these medications can result in severe deterioration of both motor and non‐motor symptoms.^2^, ^3^ It is also recommended that anti‐Parkinsonian drugs should not be withdrawn abruptly to avoid life‐threatening comorbidities such as neuroleptic malignant syndrome and Parkinsonism hyperpyrexia syndromes.^4^, ^5^, ^6^

Medication management is often challenging in patients with PD who undergo surgery. Although most PD guidelines recommended continuing oral medication even during perioperative periods,^4^, ^5^, ^6^ interruption of oral medication is often unavoidable, particularly when patients undergo gastrointestinal surgeries. This is because surgeons empirically and conventionally prefer keeping patients “nil by mouth” during perioperative periods. Therefore, patients are often required to skip several routine medications. One retrospective study of 89 surgeries in 67 patients with PD revealed that 62% of the surgeries resulted in medication withholding durations ranging from 10 to 20 h, and 17% of the surgeries resulted in withholding times of more than 20 h.^7^ In addition, patients with PD often have decreased gastrointestinal peristalsis and are predisposed to postoperative ileus, delayed gastric emptying, and vomiting, which would result in impaired bioavailability of anti‐Parkinsonian drugs. Therefore, alternative use of parenteral medications is likely to be effective during the perioperative periods.^8^, ^9^, ^10^

However, there are no established protocols for the administration of parenteral PD medications for missing doses of oral anti‐parkinsonism drugs. Some experts have recommended the use of continuous subcutaneous injection of apomorphine or the transdermal use of rotigotine.^8^, ^9^, ^10^ Nevertheless, there is only anecdotal evidence to support their efficacy in improving surgical outcomes or preventing exacerbation of symptoms and neuroleptic malignant syndrome. Furthermore, there is no consensus on which parenteral medication should be used.

A possible parenteral anti‐Parkinsonian drug candidate is intravenous levodopa (IV levodopa). Its safety has been established,^11^ and it has been generally used in experimental settings rather than in real‐world clinical settings in many countries. In fact, the clinical use of IV levodopa is approved in only a limited number of countries including Japan, therefore the clinical efficacy of IV levodopa remains unclear. Although the Japanese guidelines for PD recommend the administration of IV levodopa (Dopaston®, Ohara Pharmaceutical Co., Ltd.) as an alternative drug for the interruption of oral intake at a dose of 50–100 mg per 100 mg of oral levodopa intake,^5^ there is insufficient evidence to support the effectiveness of IV levodopa as an alternative for oral use during the perioperative period.

In the present study, we hypothesized that the use of IV levodopa can prevent perioperative deterioration of parkinsonism, leading to improved outcomes after abdominal surgery in patients with PD. To test this hypothesis, we inspected a large inpatient database in Japan to examine the efficacy of IV levodopa among patients with PD who underwent gastric or colorectal cancer surgery, in which perioperative “nil by mouth” is more likely to be applied and the duration of oral medication withdrawal were presumed to be longer than other surgeries.

## Methods

### Data Source

This was a retrospective cohort study using data from the Japanese Diagnosis Procedure Combination database.^12^ The database contained data on inpatient administrative claims and discharge abstracts from more than 1200 hospitals, accounting for almost 50% of all acute care inpatient data in Japan.^13^ The database includes the main diagnoses, comorbidities present at admission, and comorbidities occurring after admission for each patient, which were recorded both with the International Classification of Diseases and Related Health Problems 10th Revision (ICD‐10) codes and text data in Japanese. It also contained the following patient information: age, sex, body height and weight, smoking status, cancer stage, Barthel index, prescription records, treatment records, discharge status, and hospital code.

### Patient Selection

We identified patients with PD aged between 40 and 90 years who underwent partial or total gastrectomy for gastric cancer (ICD‐10 code, C16) or colorectal resection for colorectal cancer (C18‐20) who were discharged between April 2010 and March 2020. Patients with PD were identified using the ICD‐10 code for PD (G20) or prescription of anti‐Parkinsonian drugs (levodopa, levodopa carbidopa, levodopa benserazide, ropinirole, pramipexole, rotigotine, selegiline, rasagilie, and safinamide). Because the diagnoses of comorbidities in the database are known to have relatively low sensitivity,^14^ we additionally used prescription data to identify PD, as described above. Anti‐parkinsonian drugs in pharmacy records can reportedly be used as reliable markers of PD.^15^

The following patients were excluded: (i) those lacking information on habitual PD medication; (ii) those whose daily dose of anti‐parkinsonian drugs was less than 100 or more than 1500 mg/day of levodopa equivalent dose (LED),^16^ considering the possibility that they might be outliers and distort the statistical analyses; (iii) those who underwent both gastric and colorectal resection on a single admission; (iv) those who were hospitalized for the second or more times for cancer treatment; (v) those with diagnoses of secondary Parkinsonism (G21); and (vi) those who received a rotigotine transdermal patch or apomorphine injection (because they might have had additional parenteral effects during the disruption of oral intake).

### Ethical Considerations

The Institutional Review Board of The University of Tokyo approved this study and waived the requirement for informed consent because of the anonymity of the data.

### Outcomes and Variables

The primary outcomes of this study were postoperative length of stay, in‐hospital mortality, and major complications. Major complications included surgical site infection (T793, T813‐4, and T94), peritoneum abscess (K65), sepsis (A40, A41), ileus (K56, K913), pancreatic injury (K858, K859, K868), anastomosis (T818), cardiac events (I21‐25), pulmonary embolism (I26), cerebral stroke (I60‐66), renal failure (N17), respiratory failure (J12‐18, J690, 691, J95, 96), urinary tract infection (N10 30,390), fracture (S02, S12, S22, S32, S42, S52, S62, S72, S82, S92, T02, T08, T10, T11, T12, T13), and neuroleptic malignant syndrome (R509, T883, G210). While various conditions, like acute akinesia, may be associated with neuroleptic malignant syndrome, we specifically focused on neuroleptic malignant syndrome due to the lack of corresponding ICD‐10 codes for other diagnoses.

We divided the study population into two groups according to whether IV levodopa was administered on the day of surgery (“with IV levodopa” and “without IV levodopa”). We estimated the daily dose of oral PD medication by dividing the total prescribed amount during the hospital stay by the number of days of hospitalization, based on an assumption that no major changes in PD medications would be made during hospitalization for surgical purposes. We subsequently calculated the LED using the estimated daily dose,^16^ then the LED was categorized into less than 200, 200–399, 400–799, 800–1199, and ≥1200 mg/day. Presurgical comorbidities including hypertension, diabetes, and coronary artery diseases were scored according to the updated Charlson comorbidity index.^17^ Body mass index was categorized into four groups: <18.4, 18.5–24.9, 25–29.9, and ≥30.0 kg/m^2^. Activities of daily living at the time of admission was recorded as the Barthel Index, in which a higher score indicates preserved functional capacity, was categorized into four groups: 95–100, 65–90, 25–60 and 0–20. Several reports indicated that the Barthel Index was correlated with PD severity.^18^, ^19^ Hospital volume was defined as the number of patients with PD who underwent gastric and colon cancer surgery at each hospital, and was categorized into tertiles (low, middle, and high).

### Statistical Analysis

We conducted propensity score analysis using overlap weighting to adjust for confounding factors, along with multiple imputation methods to account for missing data on body mass index, Barthel index, and cancer stage. The overlap weighting method has some advantages over other conventional propensity score methods, such as matching or inverse probability weighting, in that it can achieve an exact balance in measured variables and optimize the precision of the estimated association between treatment and outcome. The target population generated mimicked the cohort of a randomized controlled trial without excluding patients from the original cohort. Details of overlap weighting are described elsewhere.^20^, ^21^ The propensity score after multiple imputations was described and validated in a previous study.^22^ Absolute standardized differences were used to evaluate differences in patient characteristics between groups. We regarded an absolute standardized difference of <0.1 as an acceptable balance of covariates between the groups.^23^

Specifically, we prepared 20 imputed datasets using the multivariate imputation by chained equations technique^24^, ^25^ and calculated the propensity score for receiving IV levodopa in each imputed dataset by applying a generalized estimating equation. The estimated propensity score was then used for overlap weights, defined as “1–propensity score” for patients with IV levodopa and “propensity score” for those without IV levodopa, in each imputed dataset. Thereafter, we performed weighted generalized linear models for the outcomes of each dataset. We changed the continuous variables (postoperative length of stay) into natural logarithms to satisfy the homoscedasticity condition for linear regression, and estimated percentage differences and their 95% confidence interval by exp (β) − 1, where β denotes the coefficients of the linear regression models. Finally, we combined the estimates from the 20 imputed datasets using Rubin's rules to obtain a combined imputation estimate and standard error.

The variables used for multiple imputations were as follows: age, sex, surgery type, cancer location, smoking status, hospital volume, hospital type, emergency admission, admission from facilities, daily LED, postoperative length of stay, in‐hospital death, and major complications. We used the same variables in the propensity score calculation, except for postoperative outcomes, such as postoperative length of stay, in‐hospital death, and major complications.

We performed subgroup analysis based on the ratio of IV levodopa dose to LED (IV/oral dose ratio) to evaluate dose‐dependent effects of IV levodopa. Specifically, we stratified patients with IV levodopa into two groups (IV/oral dose ratio <0.5, and ≥0.5), because an IV/oral ratio ≥0.5 is recommended in the Japanese guideline for PD. We then compared the outcomes of each group with those of the group without IV levodopa using the same approach as described above.

All statistical analyses were performed using Stata/MP version 17 (StataCorp, USA). Statistical significance was set at *P* < 0.05.

## Results

### Study Population

**Figure 1:**
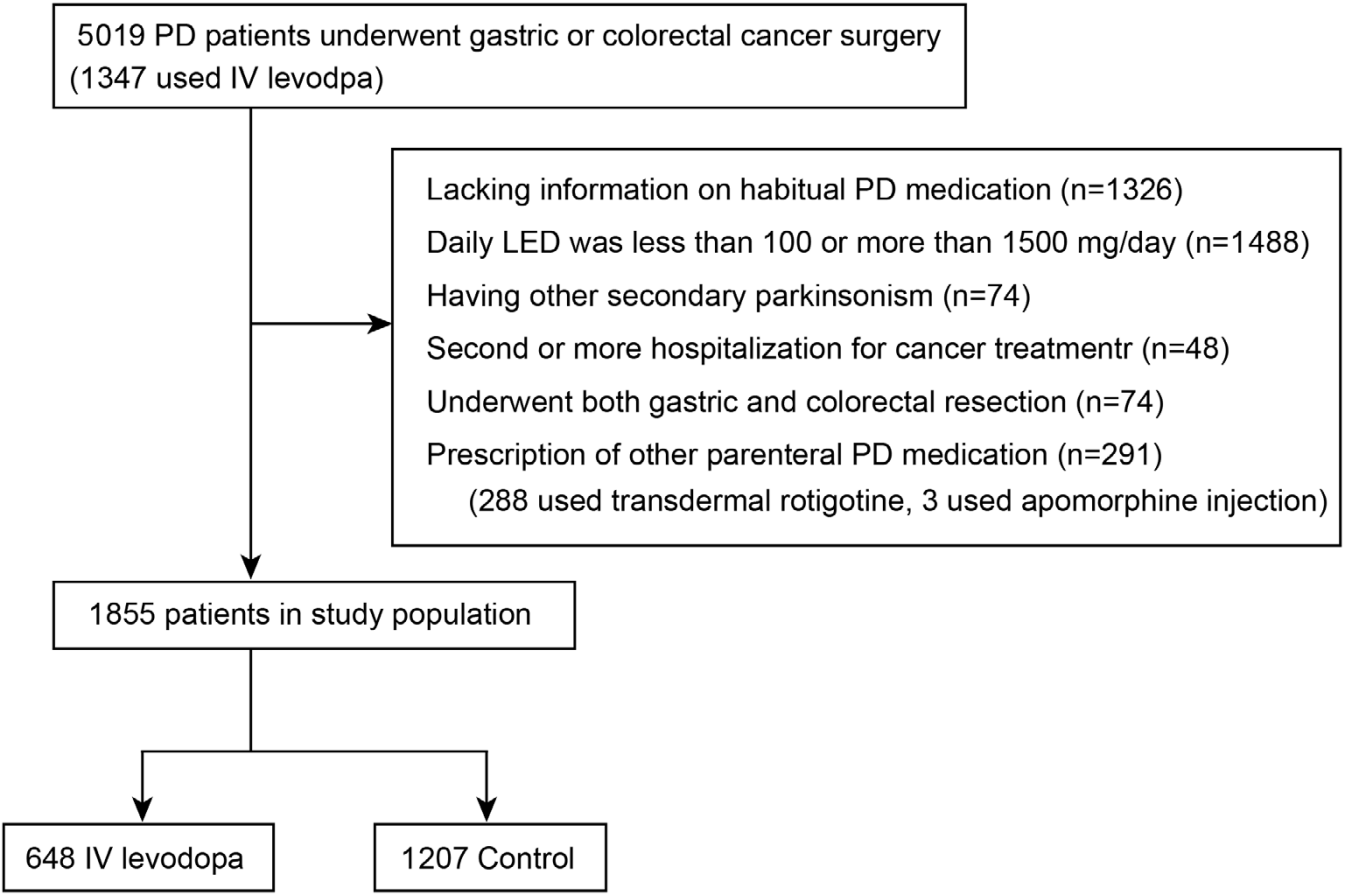
Flowchart showing patient selection.

We identified 5019 eligible PD patients who were hospitalized for gastric or colorectal cancer surgery during the study period. We excluded 3146 patients based on the exclusion criteria (Fig. 1). As a result, 648 patients who received IV levodopa during perioperative periods, and 1207 patients without IV levodopa were selected for subsequent analyses.

Table 1 shows the demographic characteristics of patients with and without IV levodopa. In the original cohort, patients with IV levodopa were significantly younger, received a larger amount of medication for PD calculated as the LED, had smaller Charlson comorbidity index scores, and were more likely to undergo surgery in larger volumes within academic hospitals. The median duration of IV levodopa administration was 4 days (interquartile range, 2–9 days). After applying multiple imputation for missing values and subsequent overlap weights, the distributions of patient characteristics were well balanced with standard differentiations of less than 0.1 in all variables, as shown in the adjusted cohort. Fig. S1 shows that the distribution of the propensity score in two groups was more balanced in the adjusted cohort.

**TABLE 1:** Demographic characteristics of the patients in the original and adjusted cohorts after multiple imputation and propensity score overlap weighting

	Original cohort			Adjusted cohort after multiple imputation and overlap weighting		
	With IV levodopa (N = 648)	Without IV levodopa (N = 1207)	SD	With IV levodopa	Without IV levodopa	SD
Age (years), %			−0.22			−0.002
40–59	1.2	0.8		0.9	1.1	
60–59	14.4	11.0		12.7	14.0	
70–79	53.7	45.7		53.9	48.2	
80–89	30.7	42.5		32.6	36.8	
Sex (female), %	42.4	44.1	−0.035	43.6	43.4	−0.005
Type of cancer, %			−0.13			−0.005
Gastric	44.1	37.6		42.1	41.8	
Colorectal	55.8	62.3		57.9	58.2	
Type of surgery, %			−0.001			−0.008
Open	54.3	53.7		54.3	53.9	
Laparoscopic	45.7	46.2		45.7	46.2	
Cancer stage, %			−0.002			−0.013
Stage I	20.8	17.1		22.4	20.5	
Stage II	36.4	39.6		40.3	40.5	
Stage III	24.2	24.9		27.2	28.6	
Stage IV	8.8	9.5		10.2	10.5	
Missing	9.7	9.0				
Smoking, %	33.9	34.6	−0.013	34.2	34.1	−0.002
Body mass index (kg/m^2^), %			0.041			−0.003
<18.5	19.4	17.5		20.1	18.0	
18.5–24.9	60.2	62.6		61.9	65.6	
25.0–29.9	14.5	15.4		16.1	14.9	
≥30.0	1.9	1.7		1.5	1.5	
Missing	4.0	2.8				
Barthel index, %			−0.068			−0.003
95–100	48.0	45.6		51.2	52.4	
65–90	14.7	12.9		15.8	14.0	
25–60	14.0	15.7		16.2	17.0	
0–20	15.1	16.3		16.8	16.6	
Missing	8.2	9.4				
Daily dose of PD medication (LED, mg), %			0.38			0.015
100–199	39.5	57.5		44.2	49.5	
200–399	41.8	32.1		41.7	34.6	
400–799	12.5	7.0		10.2	9.6	
800–1199	4.0	2.8		2.9	5.0	
1200–1499	2.2	0.7		1.0	1.3	
Charlson comorbidity index, %			**−0.14**			−0.013
0–2	73.9	66.9		72.3	71.3	
3–4	18.4	23.3		19.5	20.1	
≥5	7.7	9.8		8.2	8.1	
Emergency admission %	10.7	14.0	**−0.10**	11.3	10.9	−0.013
Admission from home, %	94.0	91.0	**0.11**	93.2	93.0	
Hospital volume, %			0.20			−0.016
Low	28.9	38.4		31.7	32.3	
Middle	33.0	30.6		34.4	31.8	
High	38.1	31.1		34.0	36.0	
Academic hospital, %	27.6	17.9	0.25	24.1	24.4	0.006

Abbreviations: IV, intravenous; LED, levodopa equivalent dose; PD, Parkinson's disease; SD, standardized difference.

*Note*: Bold indicates absolute standardised difference as 0.1.

### Main Analysis

Table 2 shows the descriptive statistics for the adjusted cohort's outcomes as well as the results of the weighted generalized linear models for the primary outcomes in the main analysis. The mean postoperative length of stay was 24.7 and 29.0 days, in‐hospital death was 3.2% and 3.3%, and major complication rate was 19.3% and 21.4% in patients with and without IV levodopa, respectively. In the weighted generalized linear models, we found a significant decrease in postoperative length of stay in patients with IV levodopa (percent difference, −7.7%; 95% confidence interval, −13.1 to −1.5), whereas in‐hospital deaths (adjusted odds ratio, 0.95; 95% CI: 0.54–1.67) and major complications (adjusted odds ratio, 0.89; 95% CI: 0.70–1.13) were comparable between the two groups.

**TABLE 2:** Postoperative outcomes in patients with and without intravenous levodopa in the adjusted cohort after multiple imputation and propensity score overlap weighting and the results of weighted generalized linear models for each outcome

Out	Adjusted cohort		Weighted generalized linear models	
	With IV levodopa	Without IV levodopa	Percent difference	*P*
Mean postoperative length of stay, days (95%CI)	24.7 (23.0–26.3)	29.0 (27.1–30.8)	−7.7 (−13.1 to −1.5)	0.011

	With IV levodopa	Without IV levodopa	Adjusted odds ratio	*P*
In‐hospital death, % (95%CI)	3.2 (1.7–4.6)	3.3 (2.3–4.4)	0.95 (0.54–1.67)	0.857
Major complications, % (95%CI)	19.3 (16.1–22.5)	21.4 (18.9–23.9)	0.89 (0.70–1.13)	0.318

Abbreviations: CI, confidence interval; IV, intravenous.

The Table S1 shows the specific diagnoses of major postoperative complications in the two groups. The number (proportion) of patients with neuroleptic malignant syndrome was 3 (0.25%) among 1207 patients without IV levodopa, while none of the patients with IV levodopa had neuroleptic malignant syndrome.

### Subgroup Analyses

Table 3 shows the results of the subgroup analyses. Like the main analysis, both patients with <0.5 and ≥0.5 IV/oral ratios were associated with decreased postoperative length of stay, whereas no association was found between in‐hospital deaths and major complications in either group.

**TABLE 3:** Subgroup analyses for postoperative outcomes and weighted generalized linear models based on the intravenous to oral dose ratio of habitual medications for Parkinson's disease in the adjusted cohort after multiple imputation and propensity score overlap weighting

		Adjusted cohort		Weighted generalized linear models	
IV/oral ratio	Outcomes	With IV levodopa	Without IV levodopa	Percent differences	*P*
IV/oral <0.5	Mean postoperative length of stay, days (95%CI)	24.3 (22.2–26.4)	29.5 (27.6–31.4)	−9.4 (−16.1 to −2.3)	0.011

		With IV levodopa	Without IV levodopa	Adjusted odds ratio	*P*
IV/oral <0.5	In‐hospital death, % (95%CI)	2.5 (0.9–4.2)	3.4 (2.3–4.6)	0.72 (0.34–1.53)	0.402
	Major complications, % (95%CI)	19.5 (15.2–23.7)	21.6 (15.3–23.7)	0.88 (0.64–1.19)	0.399

		With IV levodopa	Without IV levodopa	Percent difference	*P*
IV/oral ≥0.5	Mean postoperative length of stay, days (95%CI)	21.3 (18.9–23.6)	28.5 (26.7–30.4)	−14.2 (−22.1 to −5.5)	0.002

		With IV levodopa	Without IV levodopa	Adjusted odds ratio	*P*
IV/oral ≥0.5	In‐hospital death, % (95%CI)	3.5 (0.4–6.5)	3.5 (2.3–4.8)	0.98 (0.37–2.59)	0.973
	Major complications, % (95%CI)	16.9 (14.2–22.5)	21.3 (18.5–24.6)	0.74 (0.47–1.19)	0.217

Abbreviations: CI, confidence interval; IV, intravenous.

## Discussion

To the best of our knowledge, this is the first study to investigate the clinical efficacy and outcomes of IV levodopa use during the perioperative period of abdominal surgery in patients with PD. A strength of our study is the use of a large nationwide database and conducting strict statistical analyses to control for bias. Our study design of using propensity score overlap weighting can mimic that of randomized controlled trials. It is generally impractical to conduct randomized placebo‐controlled trials for off‐patent drugs because of their high cost. Observational studies that use real‐world data are a feasible alternative. The present study demonstrated that IV levodopa was associated with shorter postoperative hospital stays and was not associated with mortality and morbidity. These results provide novel real‐world evidence regarding the efficacy of IV levodopa during the perioperative period.

The shorter postoperative length of stay for patients with IV levodopa suggests that IV levodopa may potentially prevent perioperative deterioration of PD. Studies have shown that poorer postoperative outcomes in patients with PD may be ascribed to various manifestations of PD, including motor symptoms, dysphagia, and cognitive dysfunction.^8^, ^9^, ^10^ Although the mechanism for the postoperative deterioration of parkinsonism may be multifactorial, changing the medication during the perioperative period can be a possible factor. Additionally, the decrease in gastrointestinal peristalsis following surgery may have reduced the bioavailability of oral PD medications, possibly leading to worsening PD symptoms. IV levodopa may have had a favorable impact on these issues, accelerating postoperative recovery. Shorter hospital stays may have prevented a decline in activities of daily living due to disuse syndrome.

Meanwhile, we did not observe any improvement in mortality or morbidity with IV levodopa. Previous studies have indicated that these surgical outcomes are worse in patients with PD. A retrospective study using inpatient data from the United States reported higher incidences of aspiration pneumonia, bacterial infections, and urinary tract infections after major abdominal surgeries in patients with PD than in those without PD.^26^ We also previously showed increased morbidity after abdominal cancer surgeries in patients with PD compared to those without PD.^27^ The lack of effect of IV levodopa on mortality and morbidity may be explained by the fact that recent standard surgeries are performed safely in patients with PD. Therefore, effects of medication are undetectable due to the very low incidences of mortality and morbidity. However, IV levodopa may be effective in shortening the length of hospital stay by preventing the deterioration of Parkinsonism. Another possible explanation for the indifference in death and major complications in our study may be attributed to the recent general strategy called early recovery after surgery (ERAS) that includes shortened perioperative periods of “nil by mouth” in gastrointestinal surgery to promote early postoperative recovery.^28^, ^29^ The duration of withdrawal from oral medications may not have been sufficiently long to have an impact on mortality and morbidity.

We did not find any concerns regarding the safety of IV levodopa in this nationwide study. Since its discovery, levodopa has been used intravenously in both clinical and experimental settings and its safety has been demonstrated for several decades.^11^ Although it has not been approved by the Federal Drug and Food Administration, the clinical use of IV levodopa has been approved in Japan since 1971. The Japanese guidelines for PD recommend its use as an alternative drug when oral administration is not possible.^5^ According to a systematic review, IV use of levodopa has been documented in at least 142 original articles comprising 2760 patients worldwide in both clinical and experimental settings, and has been shown to be as safe as oral administration.^11^

The optimal dose of IV levodopa remains unknown. Although Japanese PD guidelines recommend an IV dose between half and equivalent to oral levodopa intake,^5^ the actual IV doses used in our study cohort were less than half of the oral dose in many patients (ie, IV/oral ratio <0.5). This may be because the officially approved dose of IV levodopa in Japan was only 25–50 mg/day until 2020. The group with an IV/oral ratio <0.5 also demonstrated a shorter hospital stay, suggesting that a lower dose was similarly effective for alternative parenteral use. The oral bioavailability of levodopa in elderly patients with PD was reported to be ~85% and 63% in the presence and absence of carbidopa, respectively.^30^ Other study indicated the oral bioavailability of levodopa was only 33%,^31^ and its absorption of levodopa was reduced by dietary intake.^32^

Our study also showed that neuroleptic malignant syndrome was observed only in patients who did not receive IV levodopa. Neuroleptic malignant syndrome is a potentially fatal condition characterized by fever, altered consciousness, extrapyramidal and autonomic symptoms. One possible cause is believed to be the sudden blockage of dopamine receptors in the basal ganglia.^33^ Interruption of oral PD medication is a risk factor for neuroleptic malignant syndrome, and parenteral anti‐parkinsonian drugs during the interruption period may prevent this condition.^34^ However, the incidence of neuroleptic malignant syndromes was quite low, and the total number of events was small, even in our study using a large nationwide database. Although neuroleptic malignant syndrome was not observed in the IV levodopa group, it is difficult to draw firm conclusions on its preventive effect from the current study alone.

This study had several limitations. First, the diagnoses of PD were based on records of ICD‐10 codes and prescriptions of anti‐parkinsonian drugs in the database and were not necessarily certified by neurologists. Second, the database does not contain information on the specific methods of IV administration of levodopa, such as the rate of IV administration or whether it was intermittent or continuous. Third, the database does not contain data on the severity of PD such as the Hoehn–Yahr stage or Movement Disorder Society Unified Parkinson's Disease Rating Scale. Instead, we used the Barthel index scores which were logged in the database; its validity and reliability has been demonstrated for many diseases including PD, and it is correlated with the duration and severity of the disease.^18^, ^19^ Fourth, the database does not contain the accurate dosages of anti‐parkinsonian medications, and therefore we had to estimate the doses from the total amount of medications prescribed daily. Furthermore, the database does not contain information on the specific profile of the side effects of IV levodopa, such as nausea and other gastrointestinal symptoms, orthostatic hypotension, headache, sleepiness, and psychiatric symptoms. Therefore, it is difficult to evaluate the tolerability, feasibility, or safety of IV levodopa. Although we acknowledge these limitations, the results of our study suggest future use of non‐oral medications in the immediate post‐operative period in PD not only in cases of scheduled surgeries but also in presence of an acute condition limiting swallowing. This warrants further investigation.

## Conclusion

IV levodopa following gastric and colorectal cancer surgery was associated with a shorter postoperative length of stay, but did not reduce mortality or morbidity.

## Author Roles

(1) Research Project: A. Conception, B. Organization, C. Execution; (2) Statistical Analysis: A. Design, B. Execution, C. Review and Critique; (3) Manuscript: A. Writing of the first draft, B. Review and Critique.

S.K.: 1A, 1B, 1C, 2A, 2B, 3A

T.J.: 1A, 1B, 2A, 2C, 3B

H.Y.: 1A, 1B, 2C, 3B

H.O.: 1C, 2C, 3B

N.M.: 1A, 1B, 2C, 3B

H.M.: 1A, 1B, 2C, 3B

A.O.: 1A, 1B, 2C, 3B

Y.S.: 2C, 3B

K.F.: 1A, 1B, 2C, 3B

T.T.: 2C, 3B

M.H.: 2C, 3B

## Disclosures

**Ethical Compliance Statement:** The Institutional Review Board of The University of Tokyo approved the study and waived the requirement for informed consent due to the anonymous nature of the data (Institutional Review Board number 3501). We confirm that we have read the Journal's position on issues involved in ethical publication and affirm that this work is consistent with those guidelines.

**Funding Sources and Conflicts of Interest:** This work was supported by grants from the Ministry of Health, Labour and Welfare, Japan (21AA2007 and 20AA2005) and the Ministry of Education, Culture, Sports, Science and Technology, Japan (20H03907). There is no conflict of interest concerning the research related to the study.

**Financial Disclosures for Previous 12 Months:** SK reports grants from the Ministry of Education, Culture, Sports, Science and Technology, Japan and honoraria from Eisai, Daiichi Sankyo, UCB, Otsuka, and Kyowa Kirin. TJ reports consigned research funding from Tsumura and honoraria from Tsumura, AstraZeneca, Sanofi, and Boehringer Ingelheim, and he belongs to the laboratory of the joint program with Tsumura. HY reports grants from the Ministry of Health, Labour, and Welfare, Japan; Ministry of Education, Culture, Sports, Science and Technology, Japan; and Japan Agency for Medical Research and Development, and honoraria from Novartis, Boehringer Ingelheim, Pfizer, AbbVie, Tsumura, Chugai, and Kissei. NM belongs to the laboratory of the joint program with Tsumura. HO reports nothing to disclose. HM reports grants from the Ministry of Education, Culture, Sports, Science and Technology, Japan. AO is a member of the Department of Prevention of Diabetes and Lifestyle‐related Diseases, which is a cooperative program between The University of Tokyo and Asahi Mutual Life Insurance Company. YS reports grants from the Ministry of Education, Culture, Sports, Science and Technology, Japan and honoraria from Daiichi Sankyo and Eisai. KF reports grants from the Ministry of Health, Labour, and Welfare, Japan, and Ministry of Education, Culture, Sports, Science and Technology, Japan. TT reports honoraria from Novartis and Sumitomo Dainippon Pharma, and grants from the Japan Agency for Medical Research and Development (AMED) and Japan Society for the Promotion of Science (JSPS). MH reports honoraria from AbbVie GK, Asahi Kasei Pharma, Eisai, Kyowa Kirin, Nippon Zoki Pharmaceutical, Ono Pharmaceutical, Otsuka Pharmaceutical, Sumitomo Dainippon Pharma, and Takeda Pharmaceutical.

## Supporting information


**Figure S1.** Kernel density plots showing the distributions of propensity scores in patients with and without intravenous levodopa in the original cohort (**A**) and the adjusted cohort after overlap weighting (**B**). The distribution of patient characteristics was more balanced between those with and without IV levodopa in the adjusted cohort.

**Table S1.** Comparison of major postoperative complications between patients with and without intravenous levodopa in the adjusted cohort after multiple imputation and propensity score overlap weighting
