# Supplementary figures and images for: Perioperative Use of Intravenous Levodopa as an Anti‐Parkinsonian Drug: A Propensity Score Analysis
Source: Mov Disord Clin Pract. 2023 Oct 13;10(11):1650–8. doi: 10.1002/mdc3.13894 (PMC10654832; doi:10.1002/mdc3.13894)

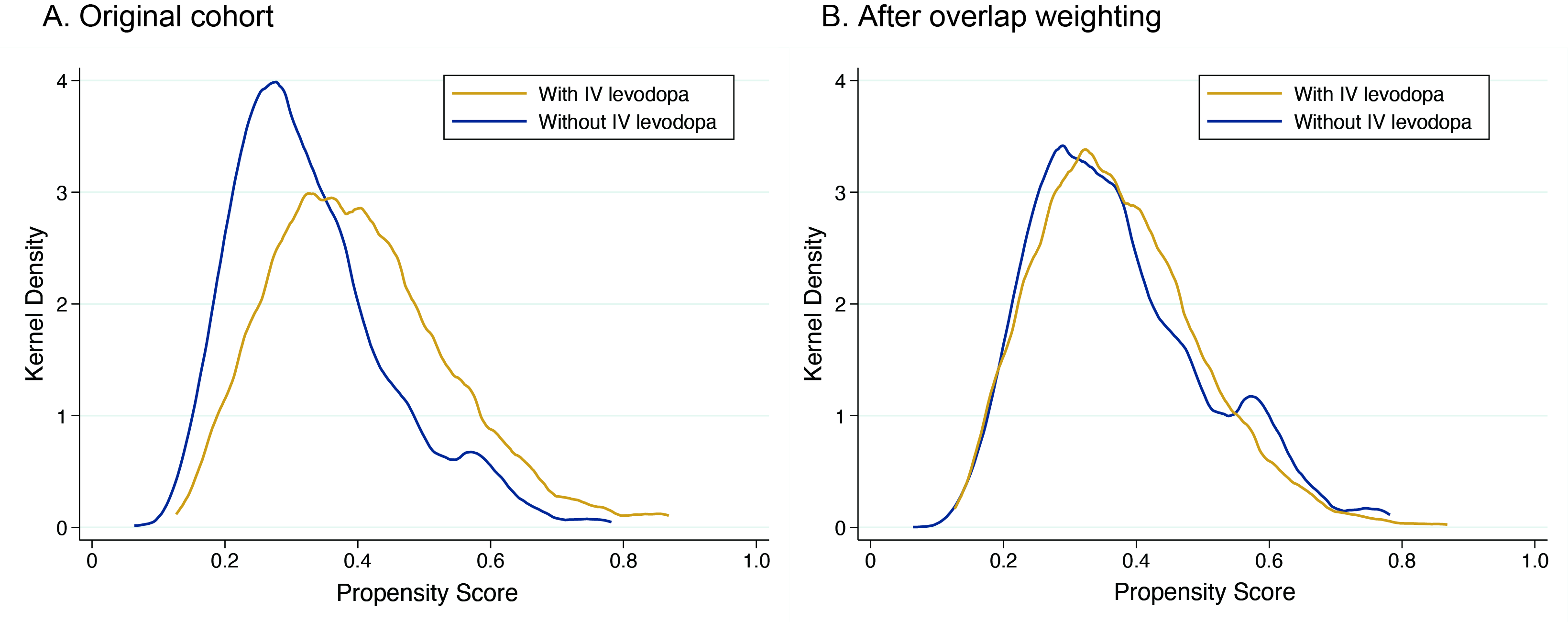

Supplement: Supplementary file 1 — Figure S1. Kernel density plots showing the distributions of propensity scores in patients with and without intravenous levodopa in the original cohort (A) and the adjusted cohort after overlap weighting (B). The distribution of patient characteristics was more balanced between those with and without IV levodopa in the adjusted cohort. [file MDC3-10-1650-s001.tif]
