# Supplementary material for: Perioperative Use of Intravenous Levodopa as an Anti‐Parkinsonian Drug: A Propensity Score Analysis
Source: Mov Disord Clin Pract. 2023 Oct 13;10(11):1650–8. doi: 10.1002/mdc3.13894 (PMC10654832; doi:10.1002/mdc3.13894)
Supplement: Supplementary file 2 — Table S1. Comparison of major postoperative complications between patients with and without intravenous levodopa in the adjusted cohort after multiple imputation and propensity score overlap weighting [file MDC3-10-1650-s002.docx]

**Supplementary Table 1**

Comparison of major postoperative complications between patients with and without intravenous levodopa in the adjusted cohort after multiple imputation and propensity score overlap weighting

|  | With  IV levodopa  (n=648) | Without  IV levodopa  (n=1,207) |
| --- | --- | --- |
| Surgical site infection, % | 3.7 | 4.4 |
| Peritoneum abscess, % | 1.9 | 1.6 |
| Sepsis, % | 1.5 | 1.9 |
| Ileus, % | 3.9 | 4.5 |
| Pancreas injury, % | 0.3 | 0.8 |
| Anastomosis, % | 7.9 | 8.8 |
| Acute coronary syndrome, % | 0.3 | 0.6 |
| Pulmonary embolism, % | 0 | 0.6 |
| Cerebral stroke, % | 0.9 | 0.7 |
| Renal failure, % | 0.6 | 0.5 |
| Respiratory failure, % | 9.1 | 8.1 |
| Urinary tract infection, % | 2.0 | 2.4 |
| Fracture, % | 0.5 | 0.5 |
| Neuroleptic malignant syndrome, % | 0 | 0.3 |

IV, intravenous.
